# Seasonal viruses modify short-term air pollution effects on pediatric wheeze and asthma: a time-series study

**DOI:** 10.1186/s12940-026-01274-y

**Published:** 2026-02-25

**Authors:** Sam Louman, Karlijn J. van Stralen, Sophie van Vlijmen, Kaj Wage, Emile Hendrix, Leontien van der Aa, Frans B. Plötz, Jeroen Hol, Anjali Kooter-Bechan, Gavin ten Tusscher, Gerard Hoek, Annemie L. M. Boehmer

**Affiliations:** 1 Department of Pediatric Pulmonology and Pediatric Allergology, Beatrix Children’s Hospital, University Medical Centre Groningen, Groningen, Netherlands; 2 Spaarne Gasthuis Academy, Spaarne Gasthuis, Haarlem, Netherlands; 3 VU University, Amsterdam, Netherlands; 4 Department of Pediatrics, OLVG, Amsterdam, Netherlands; 5 Department of Pediatrics, Rode Kruis Ziekenhuis, Beverwijk, Netherlands; 6 Department of Pediatrics, Zaans Medisch Centrum, Zaandam, Netherlands; 7 Department of Pediatrics, Blaricum, The Netherlands; 8 Department of Pediatrics and Amsterdam Reproduction & Development Research Institute, Amsterdam UMC, Location University of Amsterdam, Meibergdreef 9, Amsterdam, the Netherlands; 9 Department of Pediatrics, NWZ, Alkmaar, Netherlands; 10 Department of Pediatrics, Alrijne, Leiden, Netherlands; 11 Department of Pediatrics, Dijklander Ziekenhuis, currently De Kinderpoli NH, Hoorn, Netherlands; 12 Department of Population Health Sciences, University of Utrecht, Utrecht, Netherlands; 13 Department of Pediatrics, Spaarne Gasthuis, Haarlem, Netherlands

**Keywords:** Air pollution, Asthma, Wheeze, Children, Rhinovirus, RSV, Risk factors, Environmental health

## Abstract

**Background:**

Short-term exposure to air pollution is a known trigger for pediatric wheeze-associated disorders (WAD), such as acute asthma and virus-induced wheezing, yet few air pollution studies account for concurrent viral circulation, which may confound or modify the observed associations. This study aims to assess whether viral circulation confounds or modifies the association between daily levels of air pollution and pediatric WAD emergency department (ED) visits.

**Methods:**

We conducted a time-series analysis of 12,603 WAD ED visits among children 2–18 years old across eight hospitals in the Netherlands (2016–2023). Quasi-Poisson models estimated associations between same-day nitrogen dioxide (NO₂), fine and coarse particulate matter (PM₂․₅, PM₁₀) and ozone (O₃) and daily WAD ED visits, and adjusted for seasonality, meteorology and pollen. Base models were compared with confounding and interaction models, which included weekly rhinovirus (RV) or respiratory syncytial virus (RSV) positivity ratios as covariates or interaction terms.

**Results:**

In the base models, 3-day lag exposure to NO₂ and PM2.5 was associated with an increase in WAD ED visits (NO_2_: ER% 2.9, 95% CI 0.4—5.6; p = 0.025. PM_2.5_: ER% 3.6, 95% CI 0.4 – 6.9; p = 0.026). Associations were similar after adjustment for viral activity. The interaction models revealed attenuated effect estimates for NO_2_, PM_2.5_ and PM_10_ during relatively high RV activity. O₃ showed a negative association during low RV periods.

**Conclusions:**

In this study, circulating RV modifies, rather than confounds, the association between short-term air pollution and pediatric WAD ED visits. Considering viral activity improves the interpretation of pollution–health associations and may explain inconsistencies across studies.

**Supplementary Information:**

The online version contains supplementary material available at 10.1186/s12940-026-01274-y.

## Background

Air pollution, such as fine and coarse particulate matter (PM₂․₅, PM₁₀), nitrogen dioxide (NO_2_), ozone (O_3_), sulphur dioxide (SO_2_) and carbon monoxide (CO), has been acknowledged as a major environmental threat to human health by the World Health Organization [1]. More than 99% of the global population lives in areas where concentrations exceed the WHO guidelines (WHO, 2024). Childhood exposure to these pollutants increases asthma risk, with NO_2_ accounting for 13% of the global incidence of new pediatric asthma cases [2].

Short-term exposure to air pollutants has been consistently linked to increases in primary care visits, emergency department visits and hospitalizations for asthma and wheezing in children [3–6]. Meta-analyses reported significant associations with acute exposure to NO_2_, SO_2_, PM_2.5_, PM_10_ and O_3_ [3, 4]. In addition to air pollution, asthma exacerbations are driven largely by environmental factors, such as viral infection, with Rhinovirus (RV) infection as a primary driver [7].

Additionally, seasonal variation plays a role: in the Northern Hemisphere, asthma exacerbations peak in autumn following school return after summer holidays, and coincide with RV outbreaks, increased NO_2_ and PM from traffic and heating and decreased photochemical activity [8, 9]. Conversely, O_3_ levels are relatively high in summer because of sunlight-driven formation [10].

Air pollution studies and systematic reviews rarely account for concurrent viral circulation, despite evidence suggesting a possible interaction. Air pollutants can increase airway inflammation and oxidative stress, whereas NO₂ and PM may impair antiviral responses, enhancing rhinovirus replication [11, 12]. Epidemiologic and laboratory studies further suggest associations between air pollution and respiratory infections [13–15]. Some studies have adjusted their analyses for viral infections, such as influenza and RSV, but the nature of the interaction is rarely noted [6, 16, 17]. RV is rarely considered a covariate.

As such, the exact relationship between viral exposure concomitant with short-term air pollution exposure and pediatric wheeze associated disorder (WAD) emergency department (ED) visits is unclear. Viral circulation may act as a confounder in our region where seasonal peaks overlap, or as a modifier, where viral infection increases susceptibility to pollutants or vice versa. This study aimed to assess whether viral circulation confounds or modifies the association between daily levels of air pollution and pediatric WAD ED visits.

## Methods

### Study design

We performed a time-series study with ED visits from children from eight hospitals in the greater Amsterdam region, an area with multiple sources of air pollution, such as heavy (steel) industry, a busy international airport, urban areas and extensive road networks. The region is well covered by a network of air pollution monitoring stations providing hourly measurements [18]. This research was approved by the Institutional Review Board of Spaarne Gasthuis (2021.0065).

### Population and data collection

We included children aged 2 to 18 years who visited the emergency department of one of the eight participating hospitals between January 1 st 2016 and December 31 st 2023 for a WAD. Data were automatically extracted from the electronic health records on the basis of age, date of visit and diagnosis codes. A WAD ED visit was defined as a visit with either a registered diagnosis code for acute asthma, bronchial hyperreactivity or status asthmaticus, or if bronchodilator treatment was given during a visit with a diagnosis code for “allergic airway condition” or “unspecified lower respiratory tract condition”. Repeat visits by the same patient within 48 h were consolidated to one visit at the time of first presentation. For three of the eight hospitals, data were available from 2018 onward. Daily WAD ED visit counts were aggregated across all hospitals.

Daily levels of air pollution were obtained for NO_2_, PM_2.5_, PM_10_ and O_3_ by using hourly measurements from up to 9 background monitoring stations throughout the region [18]. A background monitoring station is not located on busy streets or at industrial sites, therefore it measures “average” levels in that particular area. The daily average level of air pollution per monitoring station was calculated by averaging the hourly levels per 24-h period per monitoring station. A daily level for the whole study region was calculated from the daily averages of all monitoring stations. A 3-day lag exposure was chosen, as this had the most consistent effect in meta-analyses [19, 20].

Data on the weekly number of positive PCR tests on swabs for a range of respiratory viruses were obtained from a regional laboratory responsible for viral diagnostics in both children and adults for hospitals, general practitioners and public health offices in the region (Streeklab, Haarlem). Approximately 85% of the virus test data is from two hospitals also providing visit data in this study, with approximately 90% of virus test data originating from adults and 10% from children. Samples were taken from a variety of respiratory sources, including the nose, throat and sputum, and tested via combined PCR and multiplex ligation-dependent probe amplification (MLPA). To approximate the level of viral circulation, we calculated a weekly test positivity rate for individual viruses by dividing the number of positive tests by the total number of tests performed for that virus, thereby adjusting for changes in the testing regime, such as during the COVID period. Based on data inspection, coronavirus (not SARS-CoV-2), influenza type A, parainfluenzavirus, rhinovirus and respiratory syncytial virus were selected as candidates for meaningful analysis (see sFigure 1). A univariate analysis was conducted to assess the relationship between weekly test positivity ratios and daily visits. RV and RSV were selected for further analysis based on associations with a p-value less than 0.1 (sTable 1).

Daily data on temperature, humidity and precipitation were obtained from a centrally located meteorological station (Royal Netherlands Meteorological Institute weather station number 240, located at Schiphol Airport). Data on daily levels of pollen were obtained from the monitoring station at Leiden University Hospital, which is located within the region. Daily totals for aeroallergenic pollen were calculated by summing the aeroallergenic subspecies in the dataset. These subspecies included Betula, Corylus, Alnus, Artemisia and Poaceae.

### Statistical analysis

We described patient characteristics by mean and standard deviation or median and interquartile range. A correlation plot was created to assess for any collinearity in the data. Variables with correlation coefficients > 0.8 were not included in the same model.

#### Time-series regression modeling

To answer our objective, we fitted three time-series models for each pollutant studied.

1. A base model with pollution, seasonality (modeled via natural splines with 7 knots per year), and covariates.
2. A confounding model with the RV and RSV weekly positivity ratios included as covariates.
3. An interaction model with dichotomized viral levels (high or low based on the median) included as interaction terms with the pollutant.

Additionally, a broader lag analysis was performed with the interaction models. Lags up to 7 days were considered, as systematic reviews have indicated that the strongest associations are found within the first week of lag [19, 20].

All models were fitted using a quasipoisson regression technique [21] with a correction for seasonality by modeling natural splines with 7 knots per year and include adjustments for daily average temperature and relative humidity, pollen, day of the week and data availability per hospital [21]. The effect of air pollution is expressed as the excess risk (ER%) for daily WAD ED visits per 10 µg/m^3^ increase in daily mean air pollution concentration. ER% is calculated as ER% = (exp(β × 10) – 1) × 100, where β is the Poisson regression coefficient.

Several sensitivity analyses were conducted: 1) using same-day exposure to assess differences between immediate and delayed exposure; 2) using a dataset restricted to the pre-COVID years (2016–2020), which was warranted because of shifting seasonal trends; 3) using a restricted dataset with only visits for registered asthma diagnoses as these have higher certainty of being true asthma visits; and 4) using alternative spline knot numbers, which depend on inspection of the residuals of the fitted spline function.

#### Missing data

Missing environmental data were assumed to be missing completely at random because of intermittent failure of a monitoring station. When > 10% of the values were missing, the data were imputed using data from other stations and variables at the same hour. Missing values < 10% were not imputed, as their impact on regional daily means was considered negligible.

All analyses were performed using R studio (version 2024.12.1 + 563) and R (version 4.4.1) [22].

## Results

A total of 12603 visits were included in the analysis. The median age of the children at the time of the visits was 4 years (IQR 2–7 years) and 63.8% of the patients were male. Among all visits, 52.5% had a registered asthma diagnosis; the remainder were included because they had received bronchodilator treatment (Table 1).

**Table 1:** Patient baseline characteristics

Total (n)	12603
Site (*n*(%))	
*Alrijne-Leiden*	1593 (12.6)
*Dijklander-Hoorn*	716 (5.7)
*NWZ-Alkmaar/Den Helder*	1248 (9.9)
*OLVG-Amsterdam*	3401 (27.0)
*RKZ-IJmuiden*	1018 (8.1)
*Spaarne-Haarlem*	2001 (15.9)
*Tergooi-Hilversum*	1535 (12.2)
*Zaans MC-Zaandam*	1091 (8.7)
Sex = Male (*n*(%))	8036 (63.8)
Age (median [IQR])	4.0 [2.0—7.0]
Admitted (*n*(%))	6563 (52.1)
Diagnosis (*n*(%))	
*Asthma*	6443 (51.1)
*Status asthmaticus*	179 (1.4)
*Other*^*a*^	5981 (47.5)

*Patients with a diagnosis of Other had a different diagnosis code registered than either Asthma or Status Asthmaticus, but received bronchodilator treatment at the emergency department indicating that wheezing was present. *IQR * Interquartile Range

The total study period consisted of 2922 days, with a mean of 4.3 (SD 3.1, range 0–24) WAD ED visits per day. Mean (SD) daily levels of air pollution were: NO_2_ 19.3 (10.5) µg/m^3^, PM_2.5_ 10.0 (7.5) µg/m^3^, PM_10_ 16.3 (7.8) µg/m^3^ and O_3_ 50.5 (21.0) µg/m^3^ (Table 2).

**Table 2:** Study period characteristics

	total days = 2922
Visits (Mean (SD))	4.3 (3.1)
Daily NO_2_ (µg/m^3^, Mean (SD))	19.3 (10.5)
Daily PM_2.5_ (µg/m^3^, Mean (SD))	10.0 (7.5)
Daily PM_10_ (µg/m^3^, Mean (SD))	16.3 (7.8)
Daily O_3_ (µg/m^3^, Mean (SD))	50.5 (21.0)
Temperature (°C, Mean (SD))	11.4 (6.0)
Precipitation (mm, Mean (SD))	2.3 (4.7)
Humidity (%, Mean (SD))	79.1 (10.2)
Aeroallergenic pollen (n/m^3^, Median [IQR])	4.00 [1.00, 18.00]
HRV Positivity Rate (Median [IQR])	0.14 [0.09, 0.19]
RSV Positivity Rate (Median [IQR])	0.02 [0.00, 0.05]

*SD* Standard deviation, *mm* millimeter, *IQR* Interquartile Range, *RV* Rhinovirus, *RSV* Respiratory Syncytial Virus

**Fig. 1:**
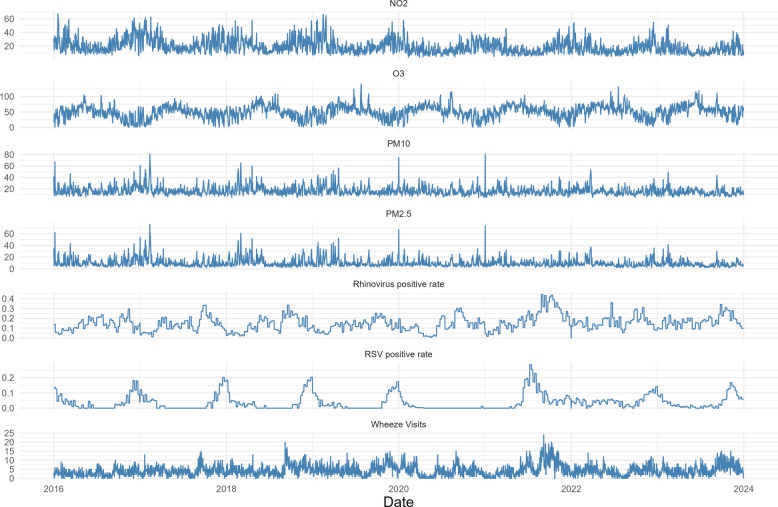
Seasonal patterns of pollutants, viruses and wheeze visits. Subscript: The y-axis represents the daily mean concentration of air pollutants in µg/m^3^, virus test positivity rate per week or daily WAD ED visits

Daily pollution, the virus positivity ratio and WAD ED visits exhibited clear seasonal patterns (Fig. 1). No correlations between covariates were > 0.8 except for daily means for PM_2.5_ and PM_10_ (sFigure 2). Plotting the residuals over time for the flexible cubic spline with 7 knots revealed some residual long-term variation, as well as signs for overcorrection, warranting sensitivity analyses using alternative knot numbers per year (sFigure 3).

**Fig. 2:**
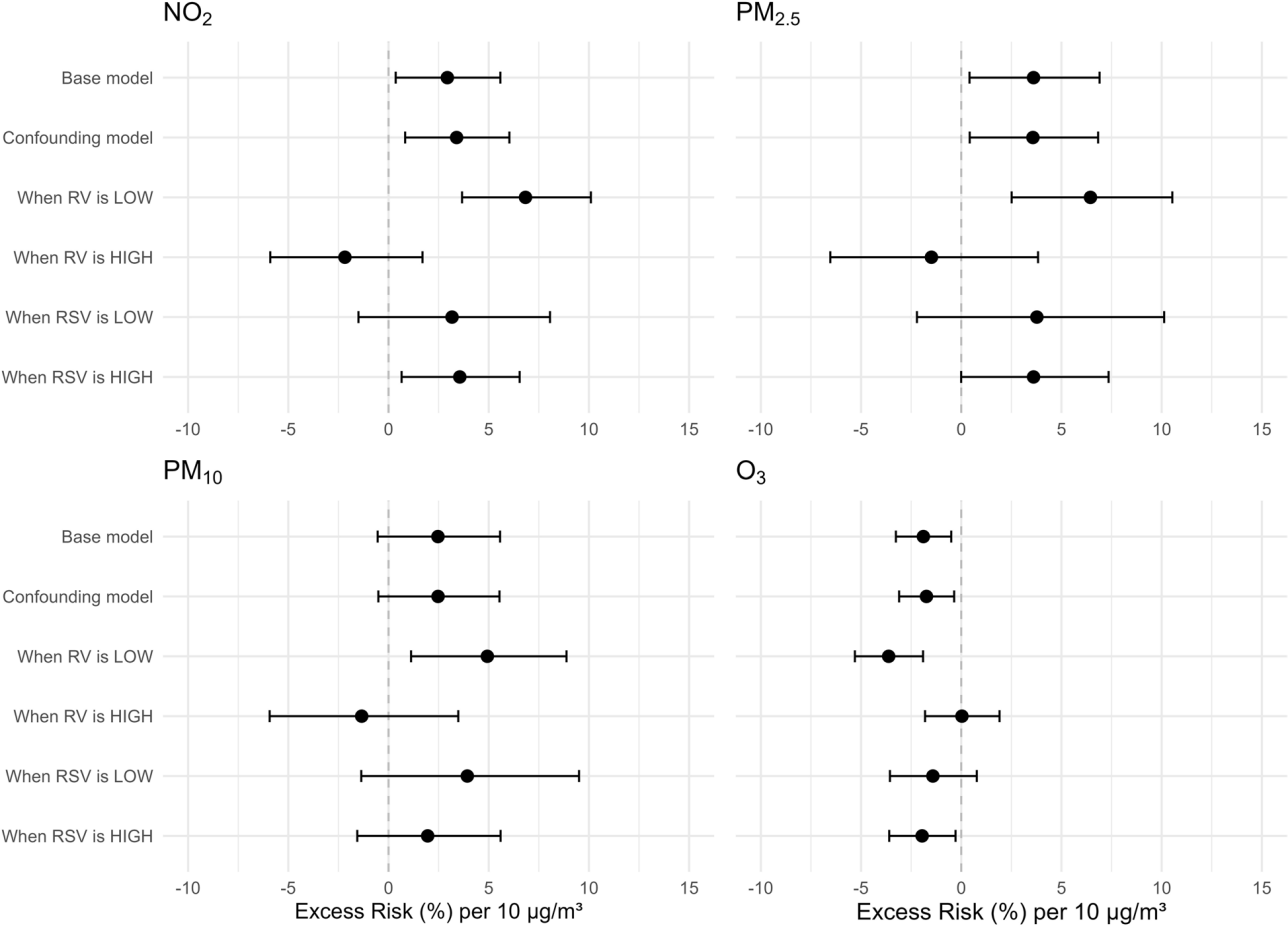
Association between 3-day lag air pollution and WAD ED visits, in all models. Subscript: Excess risks (ERs) and 95% confidence intervals for the associations between 3-day lag concentrations of air pollutants and pediatric wheeze-associated disorder (WAD) emergency visits. Models were adjusted for weather, pollen, weekday, hospital, and seasonal trends using splines. ER estimates are per 10 µg/m^3^ increase in pollutant concentration

The results of the different models for each pollutant are shown in Fig. 2 (see sTables 2–5 for outcomes in table format). In the base model, without adjustment for viral data, higher NO_2_ and PM_2.5_ were associated with more WAD ED visits (NO_2_: ER% 2.9, 95% CI 0.4—5.6; *p* = 0.025. PM_2.5_: ER% 3.6, 95% CI 0.4–6.9; *p* = 0.026). In the confounding model, adjusting for RSV and RV circulation, excess risks did not significantly change. In both models, PM_10_ did not show statistical significance. Increases in O_3_ were significantly associated with decreases in WAD ED visits in both models.

In the interaction models, with dichotomized RSV and RV levels as interaction terms, RV showed an interaction effect where air pollution showed no significant associations with WAD ED visits when RV was high, and significant associations when RV was low. High or low RSV activity did not seem to interact with the effect of air pollution on WAD ED visits. Effect estimates of continuous interaction terms showed significance for RV and not RSV, see sTable 6.

**Fig. 3:**
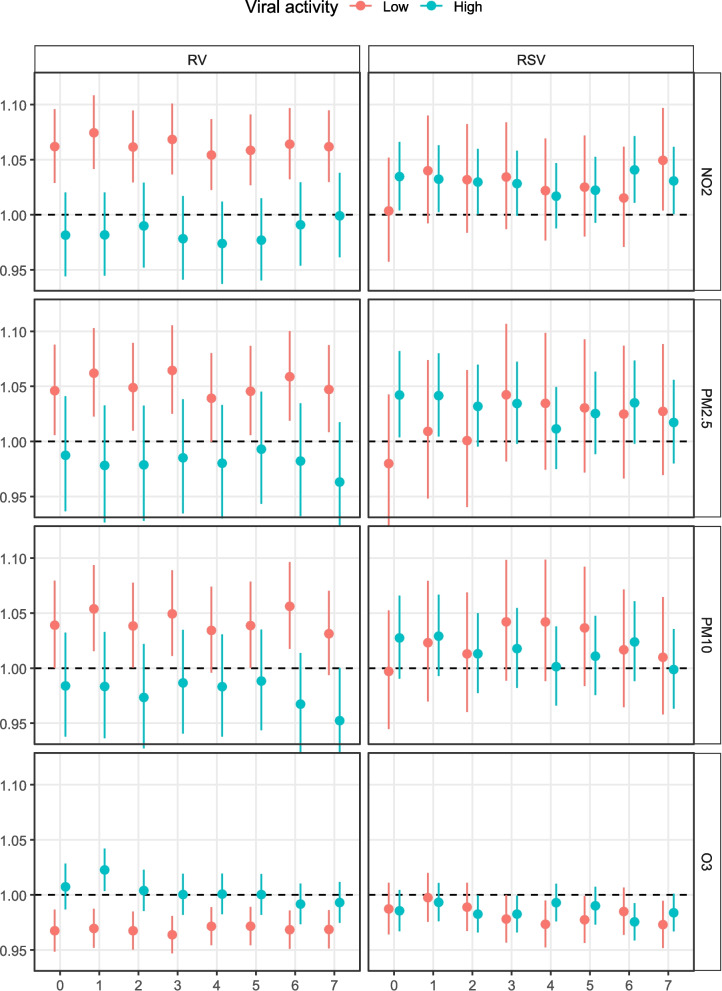
Association between air pollution exposure and WAD ED visits across 0–7 day lags, depending on viral activity level. Subscript: y-axis shows effect size per 10 µg/m^3^ increase in pollutant concentration, x-axis is lagged exposure. RV: rhinovirus. RSV: respiratory syncytial virus. NO2: nitrogen dioxide. PM2.5: particulate matter with ≤ 2.5 µm diameter. PM10: particulate matter with ≤ 10 µm diameter. O3: ozone

The results of the lagged analyses revealed similar effect sizes across lags of 0–7 days for both periods with low and high RV activity (see Fig. 3). However, for RSV activity the air pollution exposure lag chosen does affect whether an interaction effect seems present, especially for NO_2_ and PM. For example for NO_2_, choosing same-day exposure would result in finding an interaction between RSV activity and NO_2_ exposure.

Restricting the dataset to the pre-COVID years showed wider confidence intervals but no clear changes in effect direction. Restricting the population to only visits for registered asthma diagnoses yielded similar results, both with respect to effect estimates and statistical significance. The effect estimates in the sensitivity analyses using spline functions with 4 or 12 knots were consistent with those of the primary analysis, though small changes in confidence intervals occurred. Outcomes of the sensitivity analyses are shown in the Supplement.

## Discussion

We found no confounding effects, but interaction effects for RV activity, and depending on lag also for RSV activity, on the association between daily levels of air pollution and pediatric WAD ED visits. To our knowledge, this is the first study to assess this phenomenon in this way.

During periods with low RV activity, we found a statistically significant association between air pollution and more WAD ED visits, which is robust across lag periods. In periods with high RV activity, we found no statistically significant associations. Although RV is a known airway irritant [23, 24], and studies have shown that the co-occurrence of NO_2_ and PM_10_ with RV could increase viral replication [25], these findings are not supported by our data. Though speculative, a potential explanation for this paradoxical finding is that RV and pollution are competing triggers. During periods of high RV activity, the virus may be such a strong driver of WAD ED visits that any added contribution from pollution becomes difficult to observe. In other words, susceptible children may already present to the ED because of RV infection, leaving little room for an additional, detectable effect of pollution exposure. Air pollution is known to also contribute to the severity of RV induced WAD episodes [26, 27], but our study focused on visit counts rather than severity.

We did not find clear interaction effects of RSV activity on the association between 3-day lag air pollution exposure and WAD ED visits. Both RSV infection and air pollution can damage the airway epithelium [28–31]. When both are present, the greater disruption of airway function and the immune response could be expected to have a synergistic [28, 32]. In NO_2_ and PM, same-day exposure did show a possible interaction with RSV activity, where the excess risk of WAD ED visits was higher in periods with high RSV activity, potentially reflecting that effect. Whether viral infection increases susceptibility to pollutants, vice versa, or both, is not well established. In our data, we did not see clear shifts in effect estimates across lags of 0–7 days in both periods with low and high RV activity. The potential shift in interaction effects for RSV is too uncertain to make inferences on temporal relationships.

Our base model outcomes were mostly consistent with existing literature. We found significant associations between both NO_2_ and PM_2.5_ and WAD ED visits. A recent systematic review and meta-analysis revealed a significant association between 3-day lag PM_2.5_ exposure and asthma-related ED visits (2.3% increase (95% CI 0.7%—3.9) [20]. Other pollutants including NO_2_, PM_10_, and O_3_ showed nonsignificant trends between increased exposure and increased visits. Another meta-analysis reported a 4.8% (95% CI 2.8—6.7) increase in ED visits and hospitalizations for asthma in children per 10 µg/m^3^ increase in PM_2.5_ [33]. In our study, the estimated increased risk for PM_2.5_ exposure was 3.6% (95% CI 0.4—6.9) per 10 µg/m^3^. A 2017 systematic review found increased asthma exacerbations in children as a result of increased NO_2_ exposure (ER% 4.0; 95% CI 0.1–8.1), comparable to our results [34].

We also observed an inverse association for O_3_ in the base or confounding models. In the interaction models the inverse association between O_3_ and the number of WAD ED visits was more pronounced in periods of low RV activity. This suggests that during periods of low RV activity, increased O_3_ levels are associated with a reduction in WAD ED visits, which is biologically implausible. O_3_ is a known airway irritant and has often been linked to asthma exacerbations [35–37], although some studies have reported no such association [38]. The observed association is likely due to the negative correlation between O_3_ and NO_2,_ and seasonality, as NO_2_ is high when O_3_ is low and vice versa.

This study shows that it is important to consider viruses in epidemiological studies on the effects of air pollutants on childhood asthma and (viral) wheezing episodes. Not taking these factors into account may lead to incorrect effect estimates or conclusions, depending on the setting of the studies. Our findings suggest that considering viral activity in air pollution health research improves the interpretation of the health effects of air pollution in children. These findings can help elucidate the intricate interaction between air pollution and respiratory infections. Additionally, it can help disentangle and explain varying results between studies and regions, by further honing in on the true pollutant effects. Recent systematic reviews and meta-analyses do not report the incorporation of viruses in epidemiological air pollution models [34, 39], but their reciprocal interactions are acknowledged in another study [20]. Future studies should explore these interactions in other age groups and regions, and ideally incorporate individual-level viral status and exposure to air pollution data.

The strength of this study is the novel incorporation of viral surveillance data as both a potential confounder and effect modifier. The long period with real-world multicenter data and sensitivity analyses further enhance the robustness of the results. However, several limitations should be considered. First, even though we have data on over 12000 visits, the average number of events per day is 4.3, which may limit statistical power. An average of 10 events per day has been suggested as an adequate number, although evidence is lacking [21]. Second, autocorrelation between viral test data and WAD ED visits may be present in studies where test data and visit data is derived from the same population. However, only two of the eight hospitals performed their testing at the laboratory providing the viral data. Additionally, the test data is mostly derived from adult testing. This makes substantial inflation of viral activity estimates in our study unlikely. Third, we assessed exposures over lags 0–7 days, using a 3-day lag in the primary analysis. We did not model cumulative effects or apply distributed lag non-linear models. These are also common in air pollution epidemiology, and estimates could differ under those approaches. Fourth, we estimated pollution exposure using regional averages rather than individual measurements, which may have introduced exposure misclassification and likely attenuated associations. As with all time-series designs, we could not account for individual-level determinants of ED presentation. Finally, the selection of RV and RSV for further analysis was based on data availability and significant univariate associations with daily hospital visits, with a p-value threshold of 0.1. However, this approach may ignore complex interactions between viral infections, air pollution, and asthma exacerbations. Residual confounding by circulating pathogens and unaccounted for viruses may have influenced our viral activity proxies and effect estimates.

## Conclusions

Taken together, these results highlight the importance of accounting for viral circulation in epidemiological studies of air pollution and pediatric respiratory health, and suggest that viruses may be key effect modifiers of short-term pollution effects in children with wheezing. While several limitations warrant a cautious interpretation, they do not undermine the novel contribution of this study on the importance of exploring virus-pollution interactions in real-world multicenter data.

## Supplementary Information

Supplementary Material 1.

## Data Availability

The datasets used and/or analyzed during the current study are available from the corresponding author on reasonable request.
