# Supplementary material for: Seasonal viruses modify short-term air pollution effects on pediatric wheeze and asthma: a time-series study
Source: Environ Health. 2026 Feb 25;25:24. doi: 10.1186/s12940-026-01274-y (PMC13040769; doi:10.1186/s12940-026-01274-y)
Supplement: Supplementary file 1 — Supplementary Material 1. [file 12940_2026_1274_MOESM1_ESM.docx]

Supplement for: Seasonal viruses modify short-term air pollution effects on pediatric wheeze and asthma: a time-series study

Table of contents

[Supplement for: Seasonal viruses modify short-term air pollution effects on pediatric wheeze and asthma: a time-series study. 1](#_Toc221612086)

[Data inspection and univariate analysis of viruses 3](#_Toc221612087)

[sFigure 1. Data patterns of weekly positivity rates for viruses 3](#_Toc221612088)

[sTable 1. Univariate association between daily visits and weekly virus positivity rates 4](#_Toc221612089)

[sFigure 2. Correlation plot 5](#_Toc221612090)

[sFigure 3. Residuals of flexible cubic spline model with 7 knots 5](#_Toc221612091)

[Primary outcomes: tabled 6](#_Toc221612092)

[sTable 2. Association between ED visits and lag 3 NO_2_ 6](#_Toc221612093)

[sTable 3. Association between ED visits and lag 3 PM_2.5_ 6](#_Toc221612094)

[sTable 4. Association between ED visits and lag 3 PM_10_ 6](#_Toc221612095)

[sTable 5. Association between ED visits and lag 3 O_3_ 6](#_Toc221612096)

[sTable 6. Continous interaction terms outcomes 7](#_Toc221612097)

[Outcomes of sensitivity analyses 8](#_Toc221612098)

[S1: Using same-day exposure 8](#_Toc221612099)

[Figure 8](#_Toc221612100)

[Table 9](#_Toc221612101)

[S2: Restricting dataset to the preCOVID years 10](#_Toc221612102)

[Figure 10](#_Toc221612103)

[Table 11](#_Toc221612104)

[S3: Restricting the dataset to solely asthma diagnoses 12](#_Toc221612105)

[Figure 12](#_Toc221612106)

[Table 13](#_Toc221612107)

[S4: Using a spline with 4 knots 14](#_Toc221612108)

[Figure 14](#_Toc221612109)

[Table 15](#_Toc221612110)

[S5: Using a spline with 12 knots 16](#_Toc221612111)

[Figure 16](#_Toc221612112)

[Table 17](#_Toc221612113)

# Data inspection and univariate analysis of viruses

##
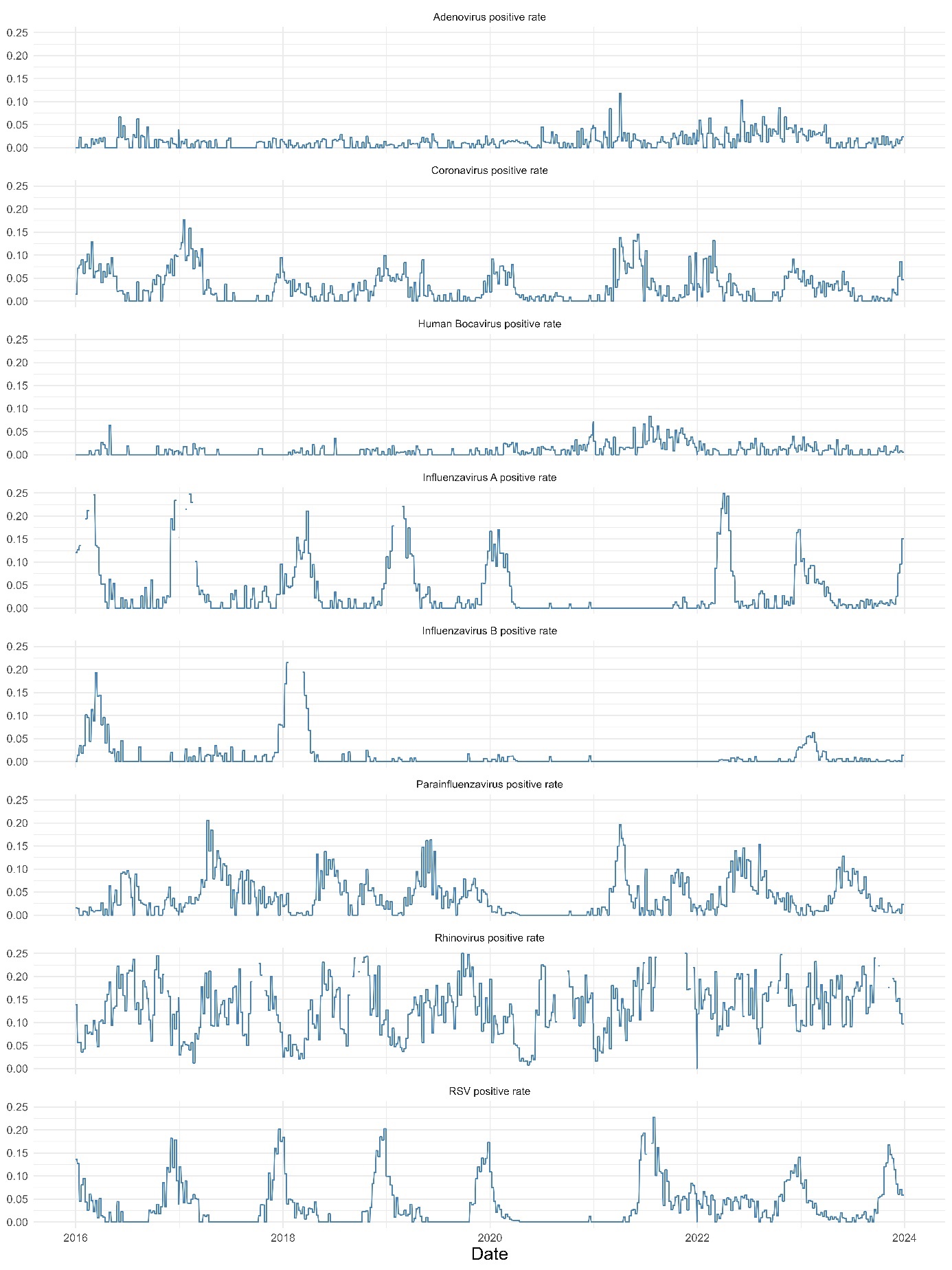
sFigure 1. Data patterns of weekly positivity rates for viruses

sFigure 1: The y-axis represents virus test positivity rate per week.

## sTable 1. Univariate association between daily visits and weekly virus positivity rates

| **Virus** | **RR (95% CI)** | **p-value** |
| --- | --- | --- |
| Coronavirus | 0.99 (0.91 – 1.06) | 0.70 |
| Influenza virus type A | 1.00 (0.96 – 1.04) | 0.96 |
| Parainfluenza virus | 0.96 (0.90 – 1.02) | 0.23 |
| RSV | 1.31 (1.26 – 1.37) | <0.001 |
| Rhinovirus | 1.36 (1.32 – 1.40) | <0.001 |

#
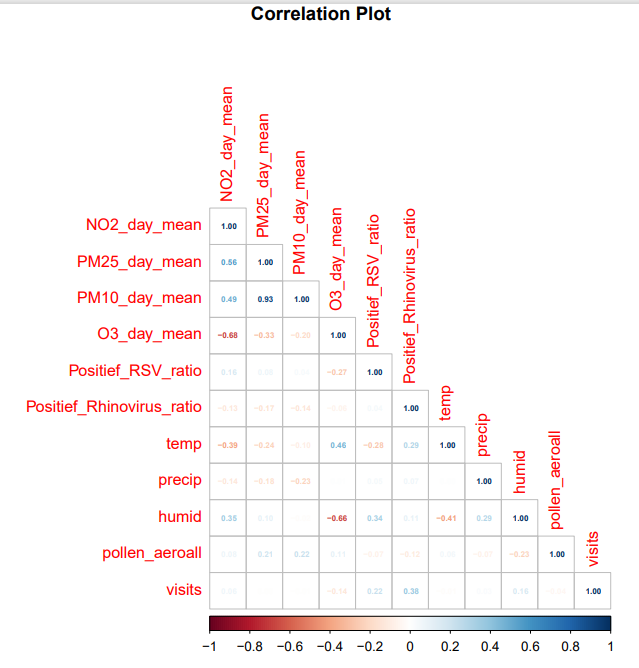
sFigure 2. Correlation plot

#
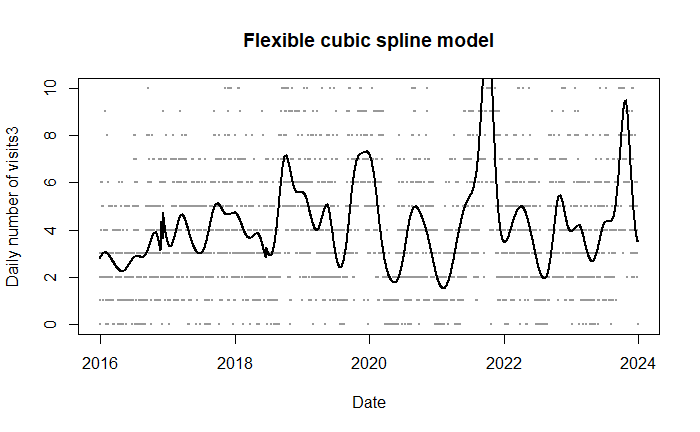
sFigure 3. Residuals of flexible cubic spline model with 7 knots

# Primary outcomes: tabled

## sTable 2. Association between ED visits and lag 3 NO_2_

| Model | Excess Risk | CI_low | CI_high | p_value |
| --- | --- | --- | --- | --- |
| BASE MODEL | 2.936 | 0.359 | 5.580 | 0.025 |
| CONFOUNDING MODEL | 3.398 | 0.830 | 6.032 | 0.009 |
| WHEN RV IS LOW | 6.836 | 3.670 | 10.099 | <0.001 |
| WHEN RV IS HIGH | -2.176 | -5.901 | 1.696 | 0.267 |
| WHEN RSV IS LOW | 3.170 | -1.498 | 8.059 | 0.186 |
| WHEN RSV IS HIGH | 3.557 | 0.654 | 6.543 | 0.016 |

## sTable 3. Association between ED visits and lag 3 PM_2.5_

| Model | Excess Risk | CI_low | CI_high | p_value |
| --- | --- | --- | --- | --- |
| Base model | 3.607 | 0.414 | 6.902 | 0.026 |
| Confounding model | 3.577 | 0.422 | 6.831 | 0.026 |
| When RV is LOW | 6.448 | 2.514 | 10.534 | 0.001 |
| When RV is HIGH | -1.487 | -6.535 | 3.833 | 0.577 |
| When RSV is LOW | 3.774 | -2.211 | 10.126 | 0.222 |
| When RSV is HIGH | 3.608 | -0.005 | 7.352 | 0.050 |

## sTable 4. Association between ED visits and lag 3 PM_10_

| Model | Excess Risk | CI_low | CI_high | p_value |
| --- | --- | --- | --- | --- |
| Base model | 2.467 | -0.542 | 5.567 | 0.109 |
| Confounding model | 2.472 | -0.506 | 5.538 | 0.105 |
| When RV is LOW | 4.932 | 1.125 | 8.881 | 0.011 |
| When RV is HIGH | -1.338 | -5.933 | 3.482 | 0.580 |
| When RSV is LOW | 3.932 | -1.360 | 9.508 | 0.148 |
| When RSV is HIGH | 1.954 | -1.559 | 5.593 | 0.279 |

## sTable 5. Association between ED visits and lag 3 O_3_

| Model | Excess Risk | CI_low | CI_high | p_value |
| --- | --- | --- | --- | --- |
| Base model | -1.890 | -3.262 | -0.500 | 0.008 |
| Confounding model | -1.736 | -3.100 | -0.354 | 0.014 |
| When RV is LOW | -3.622 | -5.308 | -1.906 | <0.001 |
| When RV is HIGH | 0.034 | -1.806 | 1.909 | 0.971 |
| When RSV is LOW | -1.416 | -3.559 | 0.776 | 0.204 |
| When RSV is HIGH | -1.953 | -3.594 | -0.284 | 0.022 |

# sTable 6. Continous interaction terms outcomes

| pollutant | virus_interaction | interaction_effect | ci_low | ci_high | p value |
| --- | --- | --- | --- | --- | --- |
| NO2 | RSV | 1.002 | 0.960 | 1.045 | 0.938 |
| NO2 | Rhinovirus | 0.949 | 0.924 | 0.976 | <0.001 |
| PM25 | RSV | 0.996 | 0.935 | 1.060 | 0.893 |
| PM25 | Rhinovirus | 0.964 | 0.930 | 0.999 | 0.043 |
| PM10 | RSV | 0.970 | 0.913 | 1.030 | 0.316 |
| PM10 | Rhinovirus | 0.966 | 0.935 | 0.997 | 0.034 |
| O3 | RSV | 0.982 | 0.959 | 1.006 | 0.136 |
| O3 | Rhinovirus | 1.020 | 1.004 | 1.035 | 0.011 |

# Outcomes of sensitivity analyses

## S1: Using same-day exposure

###
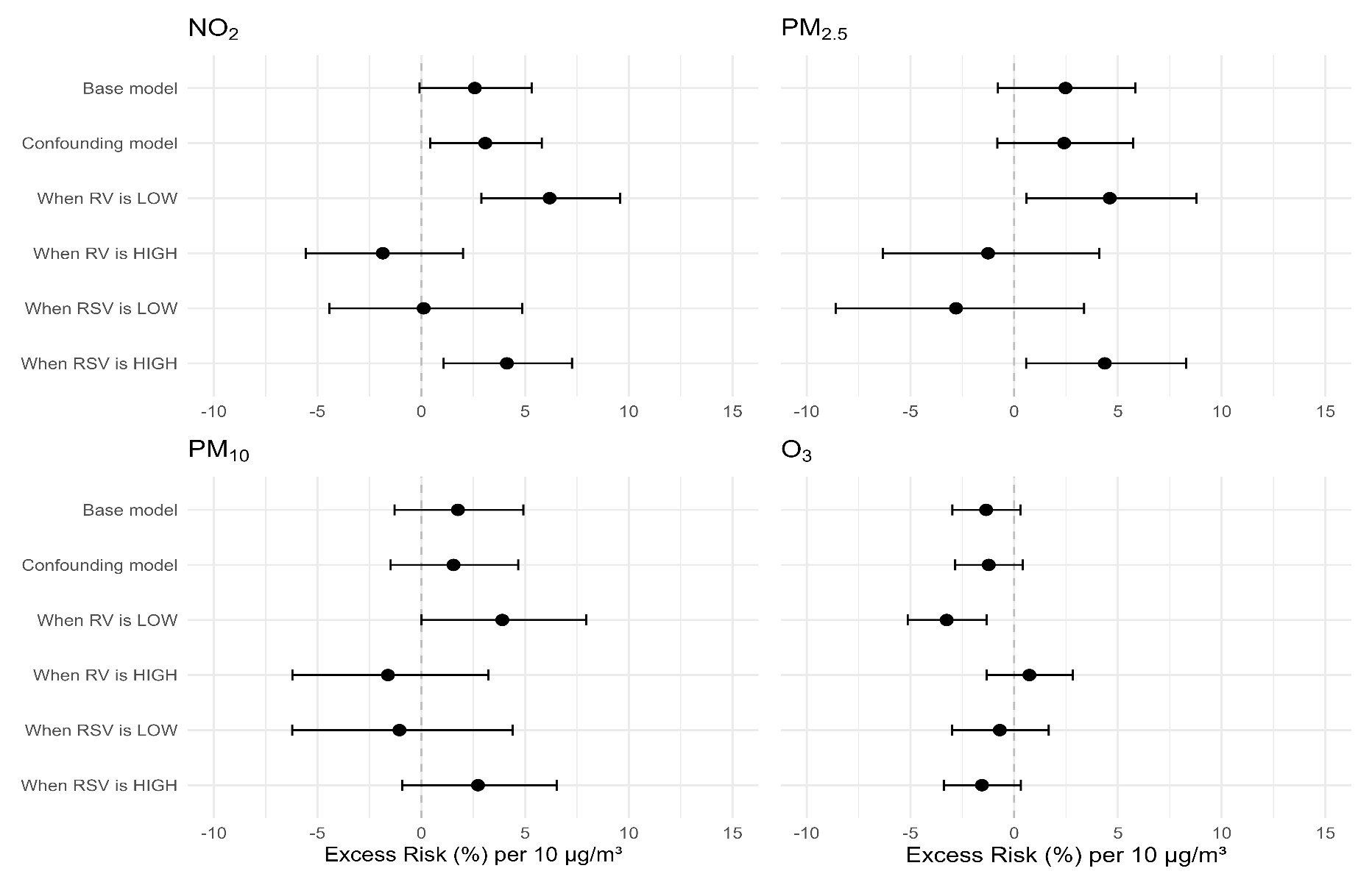
Figure

| **NO2** | | | | |  |  |  | **PM25** | | | | |
| --- | --- | --- | --- | --- | --- | --- | --- | --- | --- | --- | --- | --- |
| **Model** | **Estimate** | **CI_low** | **CI_high** | **p_value** |  |  |  | **Model** | **Estimate** | **CI_low** | **CI_high** | **p_value** |
| Base model | 2.579 | -0.087 | 5.316 | 0.058 |  |  |  | Base model | 2.475 | -0.782 | 5.838 | 0.138 |
| Confounding model | 3.083 | 0.429 | 5.807 | 0.023 |  |  |  | Confounding model | 2.409 | -0.813 | 5.736 | 0.144 |
| When RV is LOW | 6.187 | 2.895 | 9.584 | <0.001 |  |  |  | When RV is LOW | 4.607 | 0.586 | 8.790 | 0.024 |
| When RV is HIGH | -1.856 | -5.575 | 2.010 | 0.342 |  |  |  | When RV is HIGH | -1.259 | -6.336 | 4.093 | 0.638 |
| When RSV is LOW | 0.112 | -4.426 | 4.865 | 0.962 |  |  |  | When RSV is LOW | -2.805 | -8.611 | 3.370 | 0.365 |
| When RSV is HIGH | 4.125 | 1.079 | 7.263 | 0.008 |  |  |  | When RSV is HIGH | 4.364 | 0.574 | 8.297 | 0.024 |
|  |  |  |  |  |  |  |  |  |  |  |  |  |
|  |  |  |  |  |  |  |  |  |  |  |  |  |
|  |  |  |  |  |  |  |  |  |  |  |  |  |
| **O3** | | | | |  |  |  | **PM10** | | | | |
| **Model** | **Estimate** | **CI_low** | **CI_high** | **p_value** |  |  |  | **Model** | **Estimate** | **CI_low** | **CI_high** | **p_value** |
| Base model | -1.355 | -2.980 | 0.297 | 0.107 |  |  |  | Base model | 1.766 | -1.291 | 4.918 | 0.261 |
| Confounding model | -1.228 | -2.842 | 0.413 | 0.142 |  |  |  | Confounding model | 1.552 | -1.474 | 4.671 | 0.318 |
| When RV is LOW | -3.255 | -5.137 | -1.335 | 0.001 |  |  |  | When RV is LOW | 3.904 | 0.014 | 7.944 | 0.049 |
| When RV is HIGH | 0.734 | -1.320 | 2.831 | 0.486 |  |  |  | When RV is HIGH | -1.608 | -6.219 | 3.229 | 0.508 |
| When RSV is LOW | -0.695 | -3.002 | 1.666 | 0.561 |  |  |  | When RSV is LOW | -1.053 | -6.223 | 4.402 | 0.699 |
| When RSV is HIGH | -1.554 | -3.395 | 0.322 | 0.104 |  |  |  | When RSV is HIGH | 2.732 | -0.923 | 6.522 | 0.145 |

### Table

## S2: Restricting dataset to the preCOVID years

### Figure


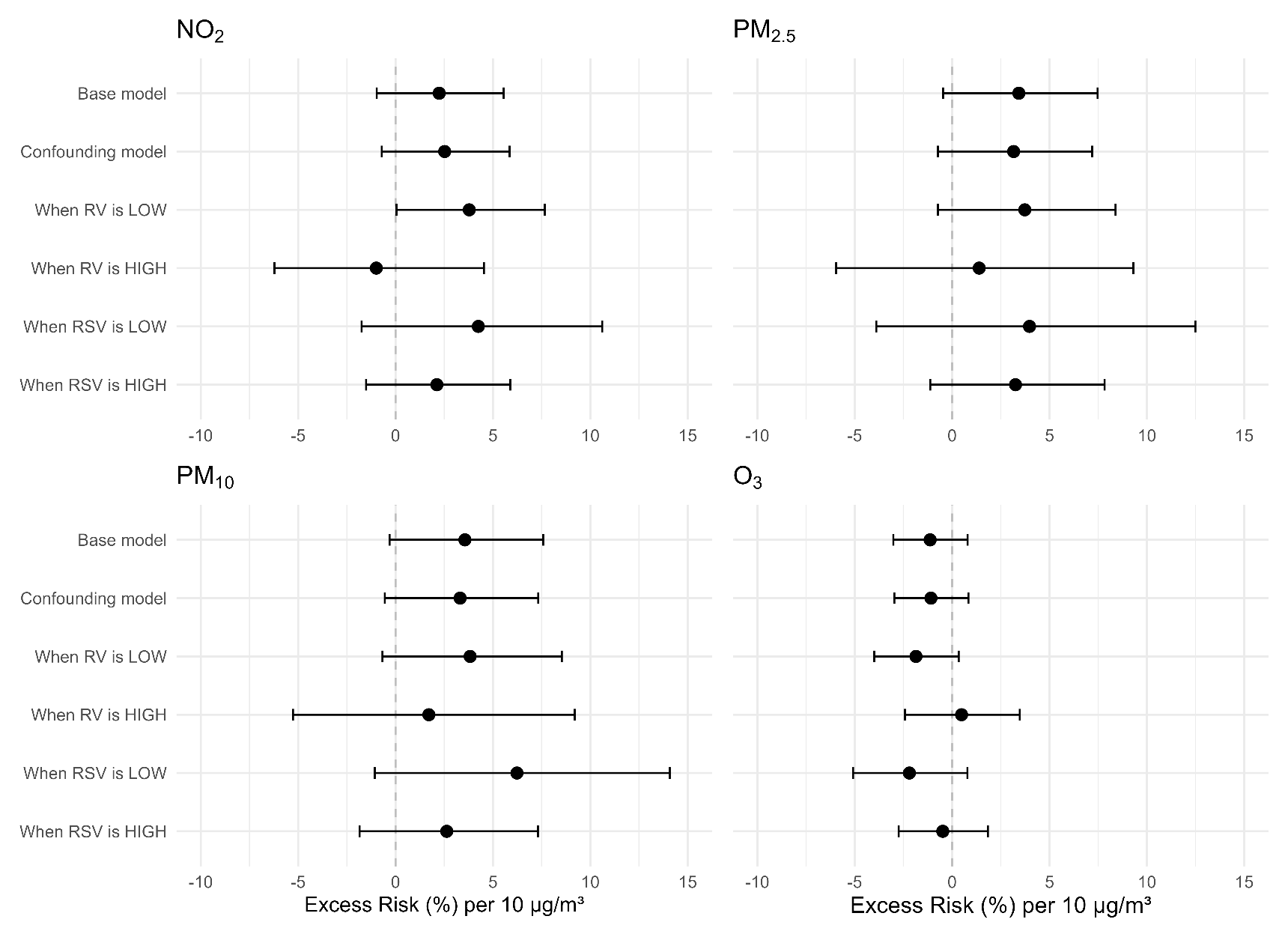


### Table

| **NO2** | | | | |  |  |  | **PM25** | | | | |
| --- | --- | --- | --- | --- | --- | --- | --- | --- | --- | --- | --- | --- |
| **Model** | **Estimate** | **CI_low** | **CI_high** | **p_value** |  |  |  | **Model** | **Estimate** | **CI_low** | **CI_high** | **p_value** |
| Base model | 2.243 | -0.968 | 5.558 | 0.173 |  |  |  | Base model | 3.428 | -0.466 | 7.475 | 0.085 |
| Confounding model | 2.523 | -0.703 | 5.854 | 0.127 |  |  |  | Confounding model | 3.160 | -0.727 | 7.198 | 0.112 |
| When RV is LOW | 3.785 | 0.048 | 7.663 | 0.047 |  |  |  | When RV is LOW | 3.733 | -0.728 | 8.395 | 0.102 |
| When RV is HIGH | -0.987 | -6.220 | 4.537 | 0.720 |  |  |  | When RV is HIGH | 1.391 | -5.951 | 9.306 | 0.719 |
| When RSV is LOW | 4.252 | -1.741 | 10.610 | 0.168 |  |  |  | When RSV is LOW | 3.975 | -3.895 | 12.490 | 0.332 |
| When RSV is HIGH | 2.123 | -1.511 | 5.891 | 0.256 |  |  |  | When RSV is HIGH | 3.255 | -1.126 | 7.829 | 0.148 |
|  |  |  |  |  |  |  |  |  |  |  |  |  |
|  |  |  |  |  |  |  |  |  |  |  |  |  |
|  |  |  |  |  |  |  |  |  |  |  |  |  |
| **O3** | | | | |  |  |  | **PM10** | | | | |
| **Model** | **Estimate** | **CI_low** | **CI_high** | **p_value** |  |  |  | **Model** | **Estimate** | **CI_low** | **CI_high** | **p_value** |
| Base model | -1.129 | -3.016 | 0.794 | 0.248 |  |  |  | Base model | 3.563 | -0.307 | 7.582 | 0.072 |
| Confounding model | -1.086 | -2.970 | 0.836 | 0.266 |  |  |  | Confounding model | 3.317 | -0.549 | 7.332 | 0.093 |
| When RV is LOW | -1.856 | -4.001 | 0.337 | 0.097 |  |  |  | When RV is LOW | 3.831 | -0.671 | 8.537 | 0.096 |
| When RV is HIGH | 0.485 | -2.422 | 3.477 | 0.747 |  |  |  | When RV is HIGH | 1.712 | -5.266 | 9.204 | 0.640 |
| When RSV is LOW | -2.193 | -5.087 | 0.789 | 0.148 |  |  |  | When RSV is LOW | 6.236 | -1.067 | 14.079 | 0.096 |
| When RSV is HIGH | -0.480 | -2.744 | 1.837 | 0.682 |  |  |  | When RSV is HIGH | 2.630 | -1.849 | 7.313 | 0.254 |

## S3: Restricting the dataset to solely asthma diagnoses

### Figure


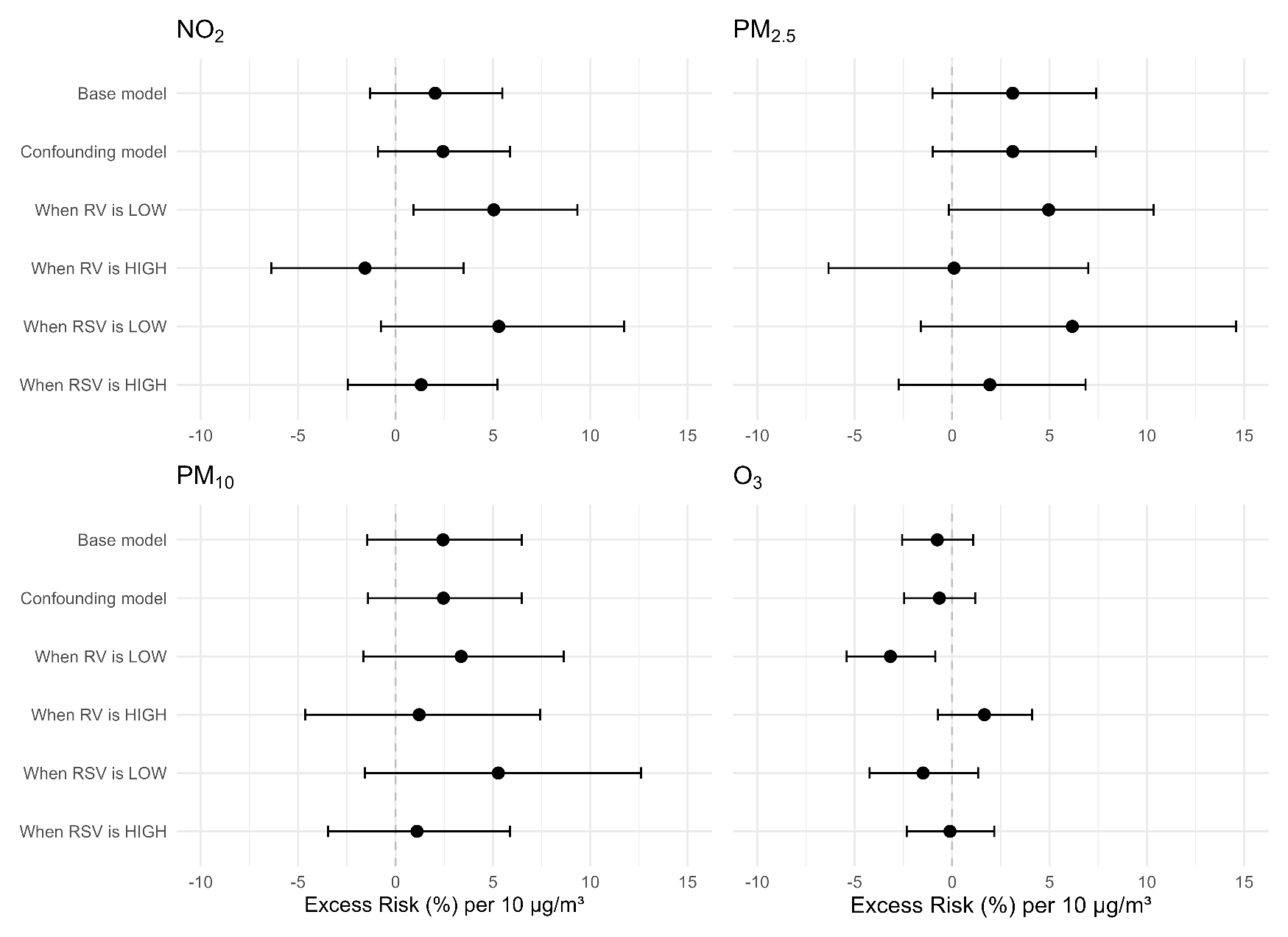


### Table

| **NO2** | | | | |  |  |  | **PM25** | | | | |
| --- | --- | --- | --- | --- | --- | --- | --- | --- | --- | --- | --- | --- |
| **Model** | **Estimate** | **CI_low** | **CI_high** | **p_value** |  |  |  | **Model** | **Estimate** | **CI_low** | **CI_high** | **p_value** |
| Base model | 2.036 | -1.298 | 5.483 | 0.234 |  |  |  | Base model | 3.109 | -1.010 | 7.400 | 0.141 |
| Confounding model | 2.434 | -0.907 | 5.888 | 0.155 |  |  |  | Confounding model | 3.112 | -0.987 | 7.380 | 0.139 |
| When RV is LOW | 5.046 | 0.923 | 9.338 | 0.016 |  |  |  | When RV is LOW | 4.957 | -0.174 | 10.351 | 0.059 |
| When RV is HIGH | -1.567 | -6.385 | 3.499 | 0.537 |  |  |  | When RV is HIGH | 0.101 | -6.349 | 6.995 | 0.976 |
| When RSV is LOW | 5.305 | -0.754 | 11.733 | 0.087 |  |  |  | When RSV is LOW | 6.175 | -1.612 | 14.579 | 0.123 |
| When RSV is HIGH | 1.317 | -2.455 | 5.235 | 0.499 |  |  |  | When RSV is HIGH | 1.937 | -2.749 | 6.849 | 0.424 |
|  |  |  |  |  |  |  |  |  |  |  |  |  |
|  |  |  |  |  |  |  |  |  |  |  |  |  |
|  |  |  |  |  |  |  |  |  |  |  |  |  |
| **O3** | | | | |  |  |  | **PM10** | | | | |
| **Model** | **Estimate** | **CI_low** | **CI_high** | **p_value** |  |  |  | **Model** | **Estimate** | **CI_low** | **CI_high** | **p_value** |
| Base model | -0.759 | -2.566 | 1.081 | 0.416 |  |  |  | Base model | 2.439 | -1.447 | 6.479 | 0.222 |
| Confounding model | -0.654 | -2.459 | 1.184 | 0.483 |  |  |  | Confounding model | 2.460 | -1.412 | 6.484 | 0.216 |
| When RV is LOW | -3.166 | -5.413 | -0.866 | 0.007 |  |  |  | When RV is LOW | 3.372 | -1.639 | 8.638 | 0.191 |
| When RV is HIGH | 1.661 | -0.724 | 4.102 | 0.174 |  |  |  | When RV is HIGH | 1.214 | -4.636 | 7.423 | 0.691 |
| When RSV is LOW | -1.49 | -4.237 | 1.335 | 0.298 |  |  |  | When RSV is LOW | 5.280 | -1.573 | 12.610 | 0.134 |
| When RSV is HIGH | -0.109 | -2.33 | 2.162 | 0.924 |  |  |  | When RSV is HIGH | 1.102 | -3.463 | 5.883 | 0.642 |

## S4: Using a spline with 4 knots

### Figure


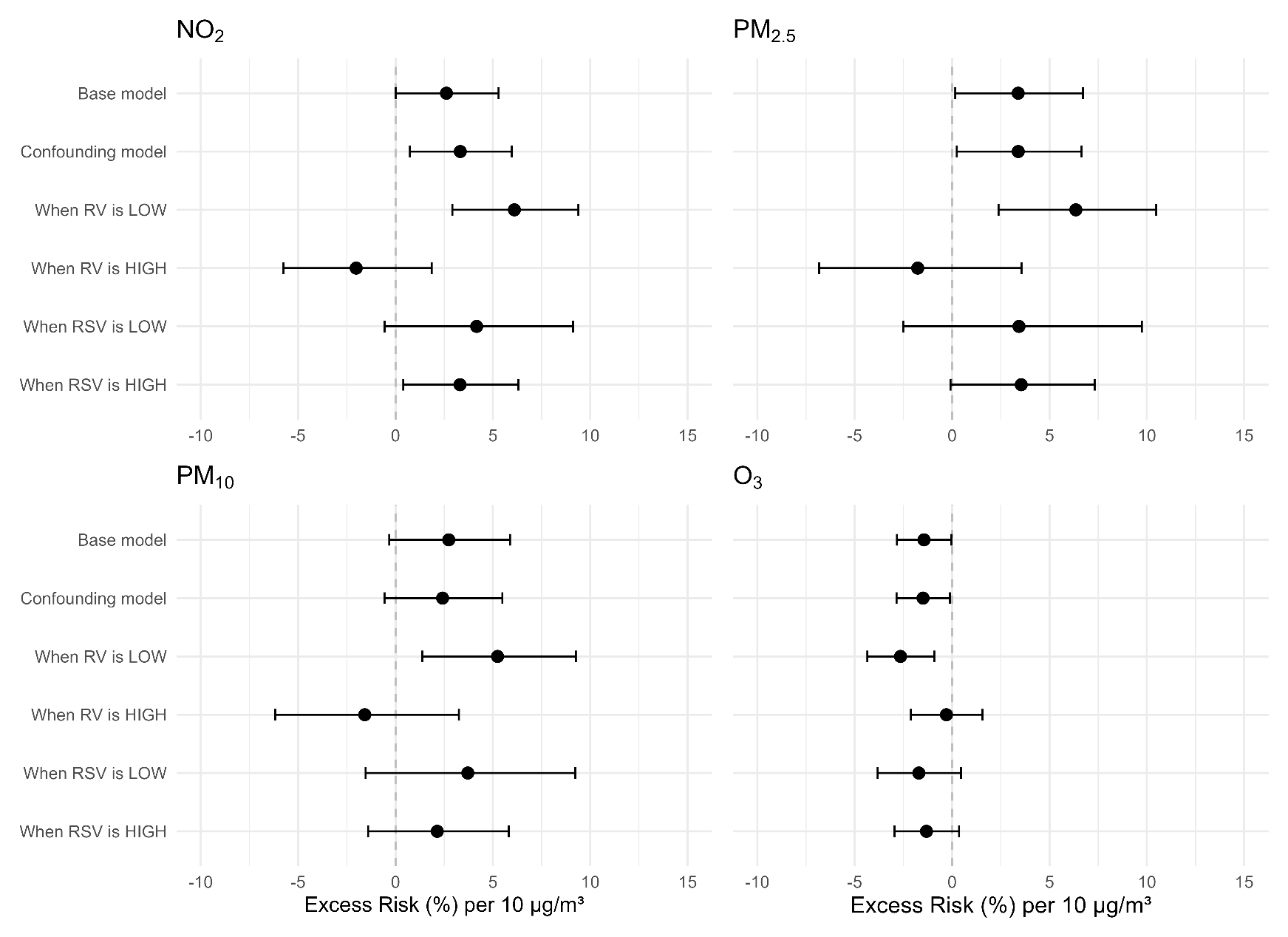


### Table

| **NO2** | | | | |  |  |  | **PM25** | | | | |
| --- | --- | --- | --- | --- | --- | --- | --- | --- | --- | --- | --- | --- |
| **Model** | **Estimate** | **CI_low** | **CI_high** | **p_value** |  |  |  | **Model** | **Estimate** | **CI_low** | **CI_high** | **p_value** |
| Base model | 2.614 | 0.000 | 5.296 | 0.050 |  |  |  | Base model | 3.393 | 0.164 | 6.726 | 0.039 |
| Confounding model | 3.325 | 0.740 | 5.977 | 0.011 |  |  |  | Confounding model | 3.396 | 0.242 | 6.650 | 0.035 |
| When RV is LOW | 6.104 | 2.917 | 9.390 | 0.000 |  |  |  | When RV is LOW | 6.354 | 2.382 | 10.480 | 0.002 |
| When RV is HIGH | -2.021 | -5.752 | 1.857 | 0.303 |  |  |  | When RV is HIGH | -1.766 | -6.822 | 3.564 | 0.509 |
| When RSV is LOW | 4.166 | -0.557 | 9.114 | 0.085 |  |  |  | When RSV is LOW | 3.434 | -2.512 | 9.743 | 0.264 |
| When RSV is HIGH | 3.313 | 0.399 | 6.312 | 0.026 |  |  |  | When RSV is HIGH | 3.549 | -0.085 | 7.316 | 0.056 |
|  |  |  |  |  |  |  |  |  |  |  |  |  |
|  |  |  |  |  |  |  |  |  |  |  |  |  |
|  |  |  |  |  |  |  |  |  |  |  |  |  |
| **O3** | | | | |  |  |  | **PM10** | | | | |
| **Model** | **Estimate** | **CI_low** | **CI_high** | **p_value** |  |  |  | **Model** | **Estimate** | **CI_low** | **CI_high** | **p_value** |
| Base model | -1.446 | -2.830 | -0.042 | 0.044 |  |  |  | Base model | 2.732 | -0.321 | 5.879 | 0.080 |
| Confounding model | -1.493 | -2.857 | -0.109 | 0.035 |  |  |  | Confounding model | 2.417 | -0.566 | 5.490 | 0.113 |
| When RV is LOW | -2.649 | -4.352 | -0.917 | 0.003 |  |  |  | When RV is LOW | 5.240 | 1.370 | 9.258 | 0.008 |
| When RV is HIGH | -0.294 | -2.115 | 1.561 | 0.754 |  |  |  | When RV is HIGH | -1.578 | -6.184 | 3.255 | 0.516 |
| When RSV is LOW | -1.707 | -3.821 | 0.453 | 0.121 |  |  |  | When RSV is LOW | 3.712 | -1.533 | 9.235 | 0.169 |
| When RSV is HIGH | -1.317 | -2.967 | 0.360 | 0.123 |  |  |  | When RSV is HIGH | 2.141 | -1.409 | 5.818 | 0.241 |

## S5: Using a spline with 12 knots

### Figure


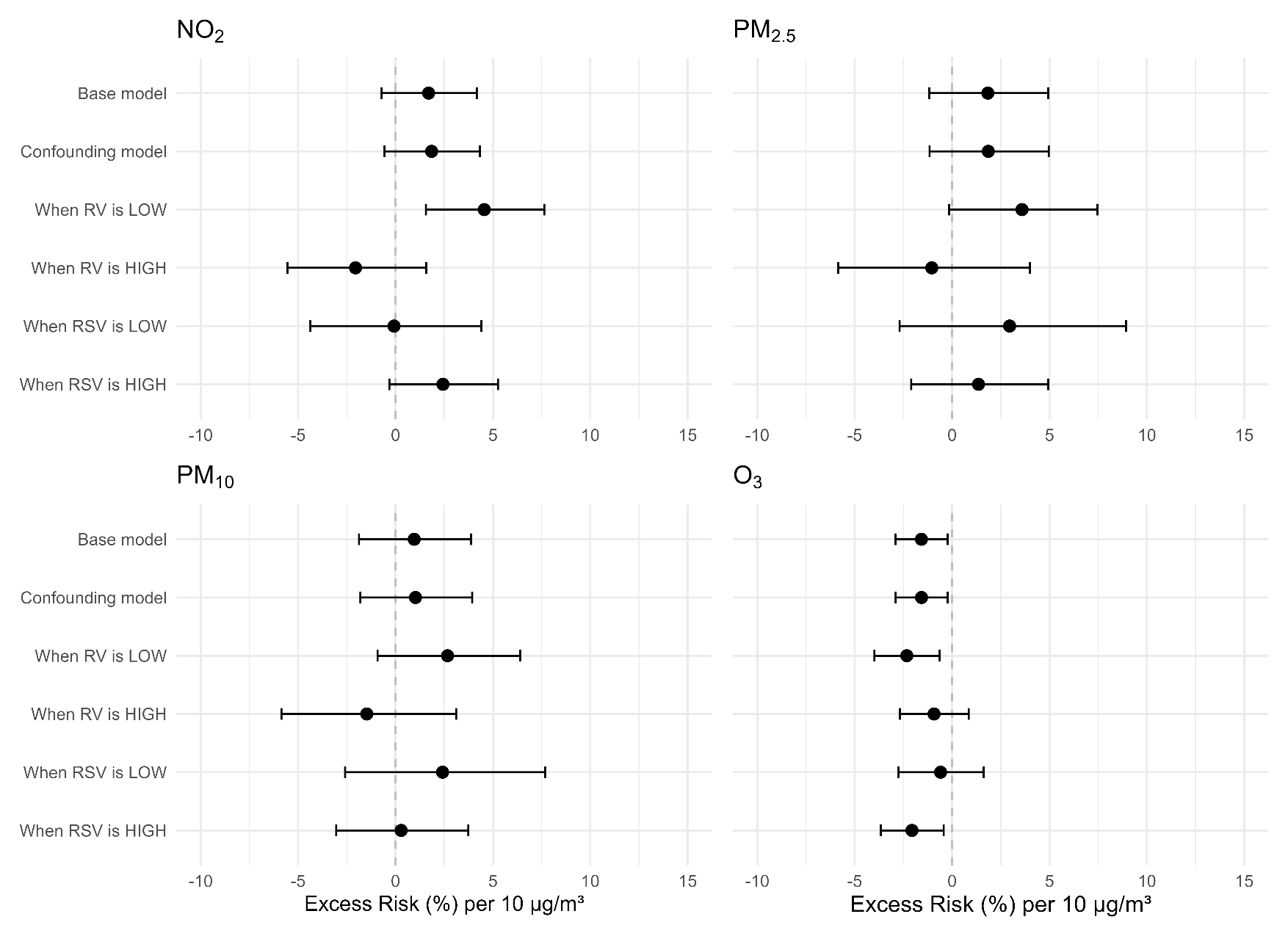


### Table

| **NO2** | | | | |  |  |  | **PM25** | | | | |
| --- | --- | --- | --- | --- | --- | --- | --- | --- | --- | --- | --- | --- |
| **Model** | **Estimate** | **CI_low** | **CI_high** | **p_value** |  |  |  | **Model** | **Estimate** | **CI_low** | **CI_high** | **p_value** |
| Base model | 1.700 | -0.714 | 4.172 | 0.169 |  |  |  | Base model | 1.837 | -1.178 | 4.943 | 0.235 |
| Confounding model | 1.853 | -0.564 | 4.328 | 0.134 |  |  |  | Confounding model | 1.858 | -1.154 | 4.962 | 0.229 |
| When RV is LOW | 4.556 | 1.561 | 7.639 | 0.003 |  |  |  | When RV is LOW | 3.590 | -0.148 | 7.469 | 0.060 |
| When RV is HIGH | -2.054 | -5.553 | 1.574 | 0.263 |  |  |  | When RV is HIGH | -1.045 | -5.850 | 4.004 | 0.679 |
| When RSV is LOW | -0.076 | -4.372 | 4.413 | 0.973 |  |  |  | When RSV is LOW | 2.952 | -2.698 | 8.929 | 0.312 |
| When RSV is HIGH | 2.433 | -0.318 | 5.261 | 0.084 |  |  |  | When RSV is HIGH | 1.355 | -2.099 | 4.931 | 0.447 |
|  |  |  |  |  |  |  |  |  |  |  |  |  |
|  |  |  |  |  |  |  |  |  |  |  |  |  |
|  |  |  |  |  |  |  |  |  |  |  |  |  |
| **O3** | | | | |  |  |  | **PM10** | | | | |
| **Model** | **Estimate** | **CI_low** | **CI_high** | **p_value** |  |  |  | **Model** | **Estimate** | **CI_low** | **CI_high** | **p_value** |
| Base model | -1.574 | -2.903 | -0.226 | 0.022 |  |  |  | Base model | 0.958 | -1.873 | 3.870 | 0.511 |
| Confounding model | -1.571 | -2.899 | -0.225 | 0.022 |  |  |  | Confounding model | 1.025 | -1.805 | 3.936 | 0.482 |
| When RV is LOW | -2.326 | -3.989 | -0.636 | 0.007 |  |  |  | When RV is LOW | 2.671 | -0.926 | 6.398 | 0.147 |
| When RV is HIGH | -0.928 | -2.673 | 0.849 | 0.304 |  |  |  | When RV is HIGH | -1.475 | -5.859 | 3.113 | 0.522 |
| When RSV is LOW | -0.592 | -2.750 | 1.614 | 0.596 |  |  |  | When RSV is LOW | 2.414 | -2.590 | 7.676 | 0.351 |
| When RSV is HIGH | -2.061 | -3.660 | -0.436 | 0.013 |  |  |  | When RSV is HIGH | 0.290 | -3.045 | 3.739 | 0.867 |
